# Real-world accuracy of robotic-assisted total knee arthroplasty and its impact on expedited recovery

**DOI:** 10.1007/s11701-024-02059-6

**Published:** 2024-08-06

**Authors:** Wai Kit Wong, Azliana Abu Bakar Sajak, Hwa Sen Chua

**Affiliations:** 1 Department of Orthopaedics and Traumatology, Hospital Ampang, Jalan Mewah Utara, Taman Pandan Mewah, 68000 Ampang, Selangor Malaysia; 2 Clinical Research Centre, Sunway Medical Centre, Bandar Sunway, 47500 Subang Jaya, Selangor Malaysia; 3 Orthopaedic Centre of Excellence, Sunway Medical Centre, Bandar Sunway, 47500 Subang Jaya, Selangor Malaysia

**Keywords:** Robotic total knee arthroplasty, MAKO, Functional alignment, Robotic surgery

## Abstract

Despite total knee arthroplasty (TKA) being the gold standard for end-stage knee osteoarthritis, 20% of patients remain dissatisfied. Robotic-assisted arthroplasty promises unparalleled control of the accuracy of bone cuts, implant positioning, control of gap balance, and resultant hip–knee–ankle (HKA) axis. Patients underwent clinical and radiological assessments, including knee CT scans and patient-reported outcome measures (PROMs), preoperatively. Follow-up assessments were conducted at 2 weeks, 6 weeks, and 3 months post-operatively, with imaging repeated at 6 weeks. A total of 155 patients underwent robotic-assisted TKA and have completed 3 months of follow-up. Mean pre-operative HKA axis was 7.39 ± 5.52 degrees varus, improving to 1.34 ± 2.22 degrees varus post-operatively. Restoration of HKA axis was 0.76 ± 1.9 degrees from intra-operative planning (*p* < 0.0005). Implant placement accuracy in the coronal plane was 0.08 ± 1.36 degrees (*p* = 0.458) for the femoral component and 0.71 ± 1.3 degrees (*p* < 0.0005) for the tibial component. Rotational alignment mean deviation was 0.39 ± 1.49 degrees (*p* = 0.001). Most patients (98.1%) had ≤ 2 mm difference in extension–flexion gaps. PROM scores showed improvement and exceeded pre-operative scores by 6 weeks post-surgery. Robotic-assisted knee arthroplasty provides precise control over traditionally subjective factors, demonstrating excellent early post-operative outcomes.

*Level of evidence* Prospective observational study—II.

## Introduction

Total knee arthroplasty (TKA) is the gold standard treatment for end-stage knee osteoarthritis (OA). However, only about 80% of patients are satisfied [1, 2]. Literature attributes dissatisfaction to issues such as component malalignment, leading to persistent pain, stiffness, or instability [2, 3]. Accurate implant positioning across the coronal, sagittal, and axial planes, along with preservation of the peri-articular soft-tissue envelope, are critical for favorable outcomes [4–6].

Alignment and soft-tissue balancing are variables under the surgeon’s control that directly influences post-operative outcome. Traditionally, component positioning has been determined intraoperatively by referencing anatomical landmarks with the aid of intra- and/or extramedullary jigs and balancing adjudged by each surgeon’s “feel” of appropriate ligament tension [8]. Technological advancements have led to the present utilization of robotic systems in our pursuit of excellence [5, 7, 9, 10].

Robotic-assisted TKA has been extensively studied, demonstrating benefits such as improved implant positioning, enhanced gap balancing, reduced soft-tissue trauma, lower post-operative pain scores, shorter hospital stays, and cost savings [2, 3, 5–7, 9–15]. With projections indicating a rise in TKA procedures in the US to 3.5 million cases by 2030, with a growing proportion under 65 years old, attention to implant survivorship is paramount [2, 9]. Consequently, significant efforts are directed toward advancing prosthetic designs, navigation technologies, and robotic systems to further refine surgical outcomes [7, 16].

This study aims to evaluate robotic-assisted TKA’s precision in implant positioning, restoration of lower limb alignment, gap balancing, and early clinical outcomes. The hypothesis posits that robotic assistance will yield more accurate implant placement, optimal lower limb alignment correction, improved gap balancing, and ultimately enhance patient outcomes within this prospective study subset.

## Materials and methods

### Patients

After receiving ethical clearance from the Sunway Medical Centre Independent Research Ethics Committee (SREC No.: 018/2023/IND/ER), all eligible patients meeting inclusion and exclusion criteria were enrolled in a prospective observational study. Inclusion criteria encompassed individuals aged 18 and above with end-stage tricompartmental knee osteoarthritis who had exhausted conservative treatments and were scheduled for robotic primary total knee arthroplasty using the Robotic Arm Interactive Orthopedic System (RIO; MAKO Stryker, Fort Lauderdale, Florida). Exclusion criteria included patients requiring revision surgery, conversion from unicompartmental knee arthroplasty (UKA) to TKA, significant bone loss necessitating additional implants or those with increased constraint implants, recent intra-articular knee injection or arthrotomy within 6 months, history of knee infection, or non-compliance with follow-up or post-operative rehabilitation programs.

### Clinical assessment

All eligible patients were then assessed on their range of motion (ROM) and knee stability before completing two patient-reported outcome measures (PROM) scoring sheets—the Knee Society Score (KSS, 2011) and the Knee Injury and Osteoarthritis Outcome Score (KOOS). This was repeated at 2 weeks, 6 weeks, and 3 months post-surgery [17]. All clinical assessments were performed by the senior author to mitigate inter-observer variability, as suggested by Johnson et al [18].

### Radiological assessment

All patients were subjected to a long-limb radiograph along with an AP and lateral view of the knee. For patients undergoing their TKA using the MAKO system (Stryker Orthopaedics, Mahwah, NJ), they required a CT scan for pre-operative planning. The same set of radiological investigations were repeated at 6 weeks post-surgery.

All radiological images were stored on a PACS system, and all measurements performed using the in-built measurement tools. Hip–knee–ankle (HKA) axis is defined by the angle subtended by a line representing the mechanical axis of the femur and another line representing the mechanical axis of the tibia. Femoral and tibial component coronal angle is measured in relation to the mechanical axis of the femur and tibia, respectively. We could not establish sagittal plane measurements for both the femoral and tibial components as we did not subject our patients to an additional long-leg lateral radiograph as this would not be the standard of care at our institution, as well as incurring additional costs. Attempts to ascertain sagittal plane alignment from the lateral knee radiographs yielded inconsistent results and do not concur with the landmarks utilized by the MAKO robotic system. Rotational alignment of the femoral component is measured on axial CT scans against the trans-epicondylar axis (TEA), which is most reproducible and has been shown to produce the best results in terms of patella tracking and eventual wear [19]. The post-operative measurements were then compared to the intra-operative values stored on the MAKO system.

### Assessment of gap balance

The values of the medial and lateral gap distance in both extension and flexion were procured from the MAKO system for analysis to determine the accuracy of gap balance restoration and the effects it had on the post-operative outcomes.

### Data analysis

Data were then analyzed using SPSS version 24 (International Business Machine Corporation; Armonk, New York, NY) with significance set at *p* < 0.05. Using central limit theorem for large sample (> 30) with equal variance as a basis, paired sample t-tests and ANOVA with repeated measures were used for comparison and Pearson’s (r) correlation was used to assess the correlation between variables [20, 21]. As for HKA grouping, Kruskal–Wallis and Mann *U* Whitney were used to determine whether degree of lower limb alignment had any significant impact on PROM scores.

The study did not conduct a separate priori power analysis given its interim nature and subset focus.

### Surgical technique

The surgical technique involved pre-operative CT scans for planning, adhering to Functional Alignment principles to achieve limb alignment restoration and gap measurements within 1 mm between flexion/extension and medial/lateral gaps [22, 23]. The surgical plan utilizes bone cuts to balance gaps instead of soft-tissue releases and is sufficient in most cases. In cases whereby bone cuts alone were not satisfactory, minimal soft-tissue releases were performed to augment the bone cuts.

After exposure using a midline skin incision and a conventional medial parapatellar approach, fiducial pins were placed on the femur and on the tibia just distal to the tibial tuberosity. The hip center along with the malleoli was then registered, followed by mapping of the distal femur and proximal tibia. The patient’s baseline limb alignment and gap measurements were recorded. Once anatomical accuracy was verified, the surgical plan for the femur and tibia was adjusted in all three planes prior to the actual bone cuts [2].

The bone cuts were completed by the surgeon, with guidance from the robotic arm. Trial implants were then used to confirm implant positioning, restoration of gap balance, and limb alignment. The cemented Triathlon Cruciate Retaining Total Knee System (Stryker Orthopaedics, Mahwah, NJ) without patellar resurfacing was utilized for all patients.

No drains were inserted for any of our patients and full-weight bearing ambulation initiated on post-op day 1 with the assistance of a 4-legged walker. Outpatient physiotherapy appointments were scheduled upon discharge to support rehabilitation.

## Results

After applying the inclusion and exclusion criteria, the study enrolled a total of 200 patients who underwent surgery at the time of writing. Fourteen patients opted out of post-operative CT scans due to concerns over additional radiation exposure, and additional 31 patients had not yet attended their post-operative 3-month assessment and were consequently excluded from this preliminary report. Thus, the final analysis included complete datasets from 155 patients.

### Demographics and pre-operative characteristics

All 155 patients had end-stage tricompartmental OA and have exhausted conservative measures. There were 103 female (66%) and 52 male patients (34%) in this study. Average follow-up duration was 9.76 months (minimum of 3 months and a maximum of 19 months). Among the 155 knees studied, 85 (55%) were right-sided and another 70 (45%) left-sided.

Average age was 66 years and 4 months, with the youngest being 46 years and oldest being 82 years. The average BMI was 26.7 kg/m^2^ with 65% of patients categorized as overweight or obese.

All patients were subjected to spinal anesthesia and average surgery duration was 66.3 ± 12.3 min. A moderately positive correlation was found between surgical duration and BMI, *r*(153) = 0.432, *p* < 0.0005, while no correlation was observed between surgical duration and degree of deformity as adjudged based on the pre-op HKA axis, *r*(153) = 0.086, *p* = 0.289.

### Range of motion

**Fig. 1:**
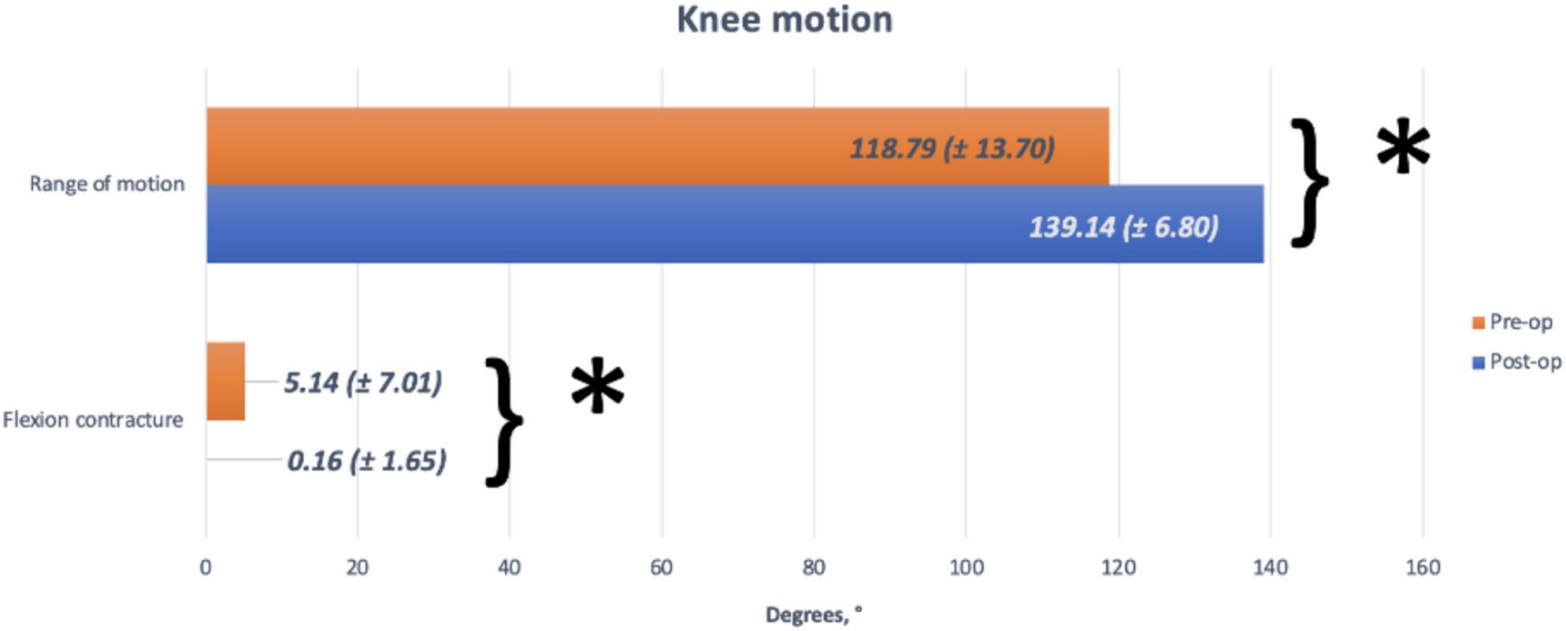
Comparison between pre-operative and post-operative range of motion and flexion contracture. *Indicates that there was a significant difference at *p* < 0.0005

Range of motion (ROM) analysis indicated that the average pre-operative ROM was 118.79 ± 13.7 degrees, with a mean flexion contracture of 5.14 ± 7.01 degrees. Post-operatively, ROM significantly improved to 139.14 ± 6.8 degrees, with nearly complete elimination of flexion contracture (*p* < 0.0005), as illustrated in Fig. 1.

### Alignment

Alignment assessments showed that the mean pre-operative HKA axis was 7.39 ± 5.52 degrees varus, which improved to a mean of 1.34 ± 2.22 degrees varus post-operatively. The restoration of the HKA axis was 0.76 ± 1.9 degrees apart from the intra-operative planned axis.

### Implant position

Regarding implant positioning, in the coronal plane, the femoral and tibial component placements deviated by 0.08 ± 1.36 and 0.71 ± 1.3 degrees, respectively, from the planned intra-operative positions. Rotational alignment deviations averaged 0.39 ± 1.49 degrees. Detailed results are summarized in Table 1.

**Table 1:** Assessment of accuracy of implant position

Comparison between intra-op & post-op component positioning	Paired differences				Significance, *p* value
	Mean, degrees (º)	Std. deviation, degrees (º)	95% Confidence interval of difference		
			Lower	Upper	
HKA^α^	0.76	1.90	0.46	1.06	0.000
Coronal femur^α^	0.08	1.36	– 0.13	0.30	0.458
Coronal tibia^α^	0.71	1.30	0.50	0.91	0.000
Rotational^α^	0.39	1.49	0.16	0.63	0.001

^α^Paired sample t test, *p* < 0.05 as significance at 95% CI

### Gap balancing

**Fig. 2:**
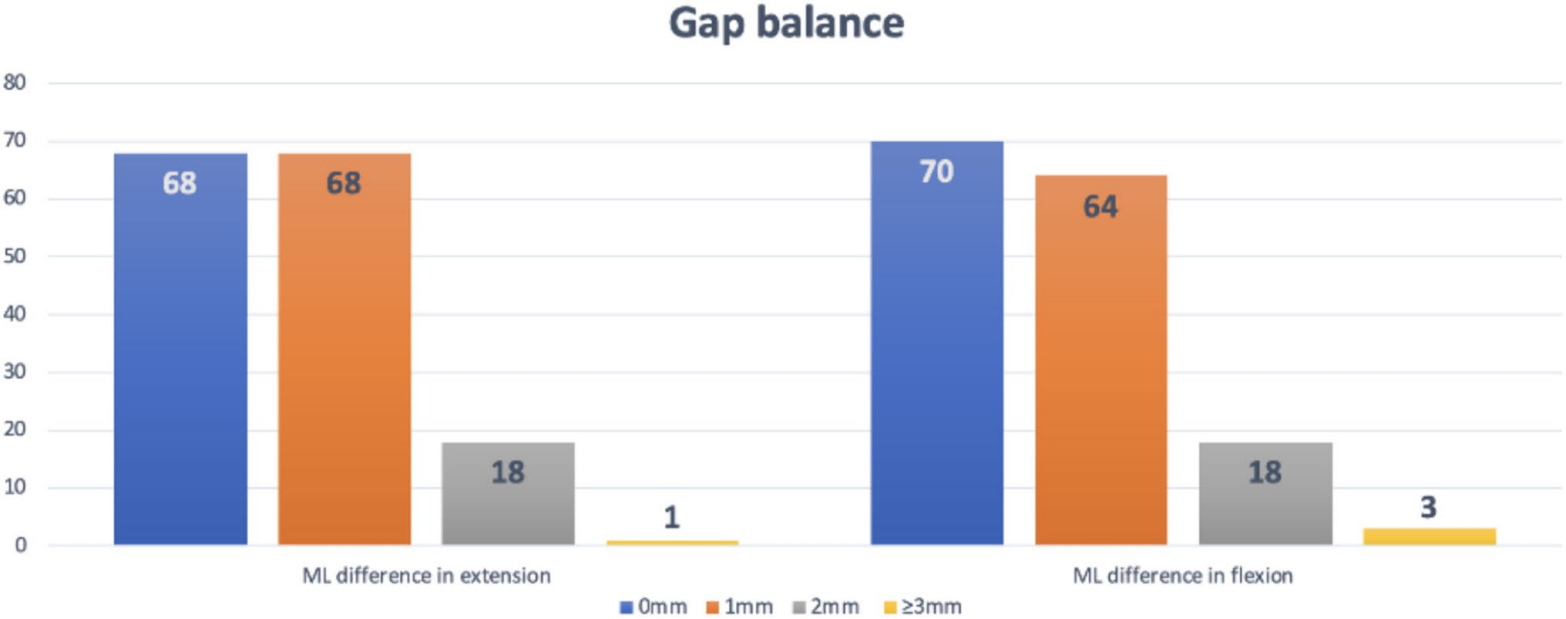
Distribution of cases according to differences between medial and lateral gaps in both extension and flexion

Gap balancing analysis revealed minimal differences, with only 1 case (0.6%) showing a medial–lateral gap difference of 3 mm or more in extension and three cases (1.9%) in flexion, as depicted in Fig. 2. The mean flexion and extension gaps were 18.36 ± 1.1 mm and 18.29 ± 1.3 mm, respectively, and the vast majority (98.1%) of knees exhibited a gap difference of 2 mm or less.

### Patient-reported outcome measures

**Fig. 3:**
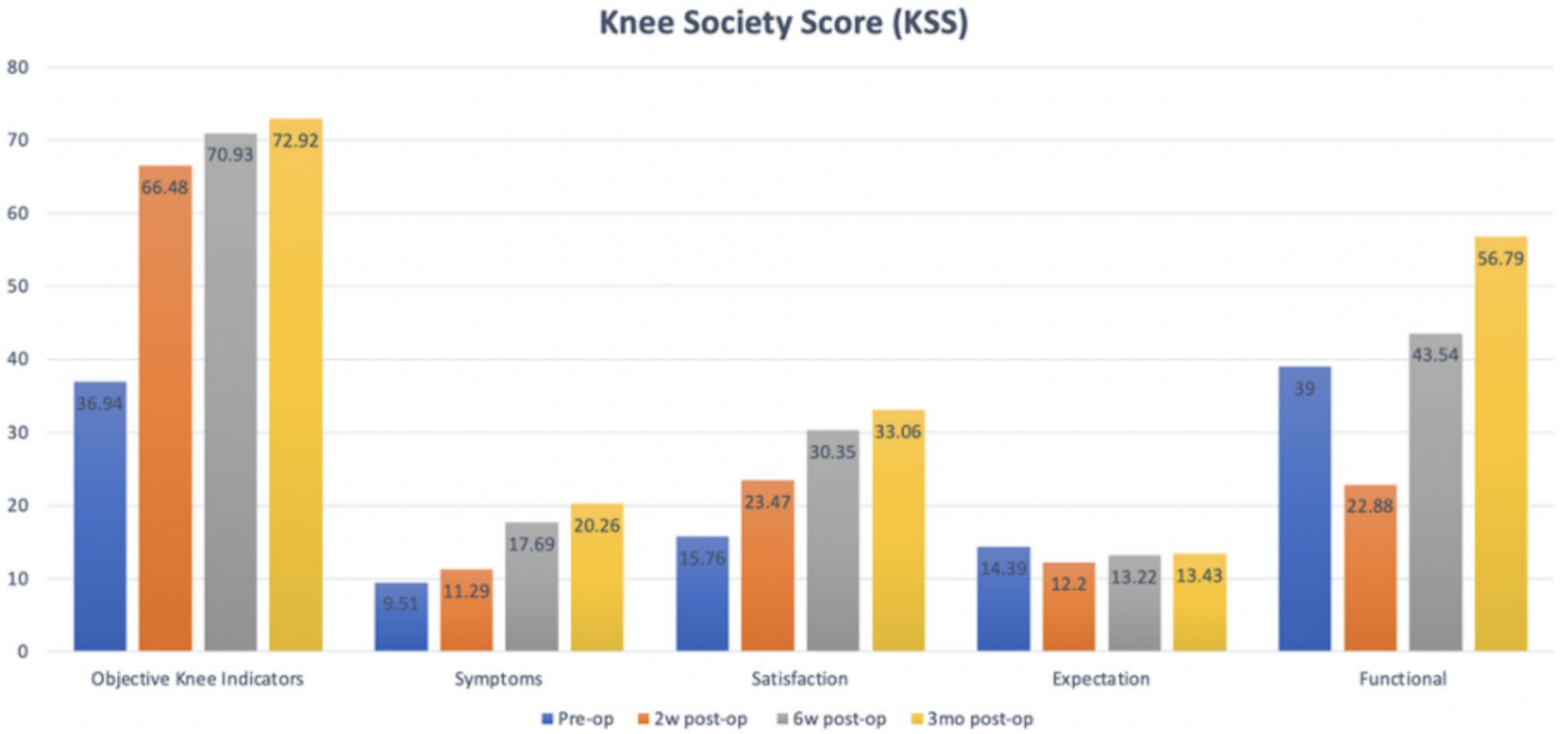
Knee Society Score distribution according to subsegment and timeline

Patient-reported outcome measures (PROMs) indicated significant improvements across various categories. Knee Society Score (KSS) subsets of Objective Knee Indicators (OKI), Symptoms, and Satisfaction improved significantly (*p* < 0.0005) by week 2 post-surgery, with further improvements observed at weeks 6 and 3 months. However, expectations remained consistent with pre-operative levels throughout, as depicted in Fig. 3.

**Fig. 4:**
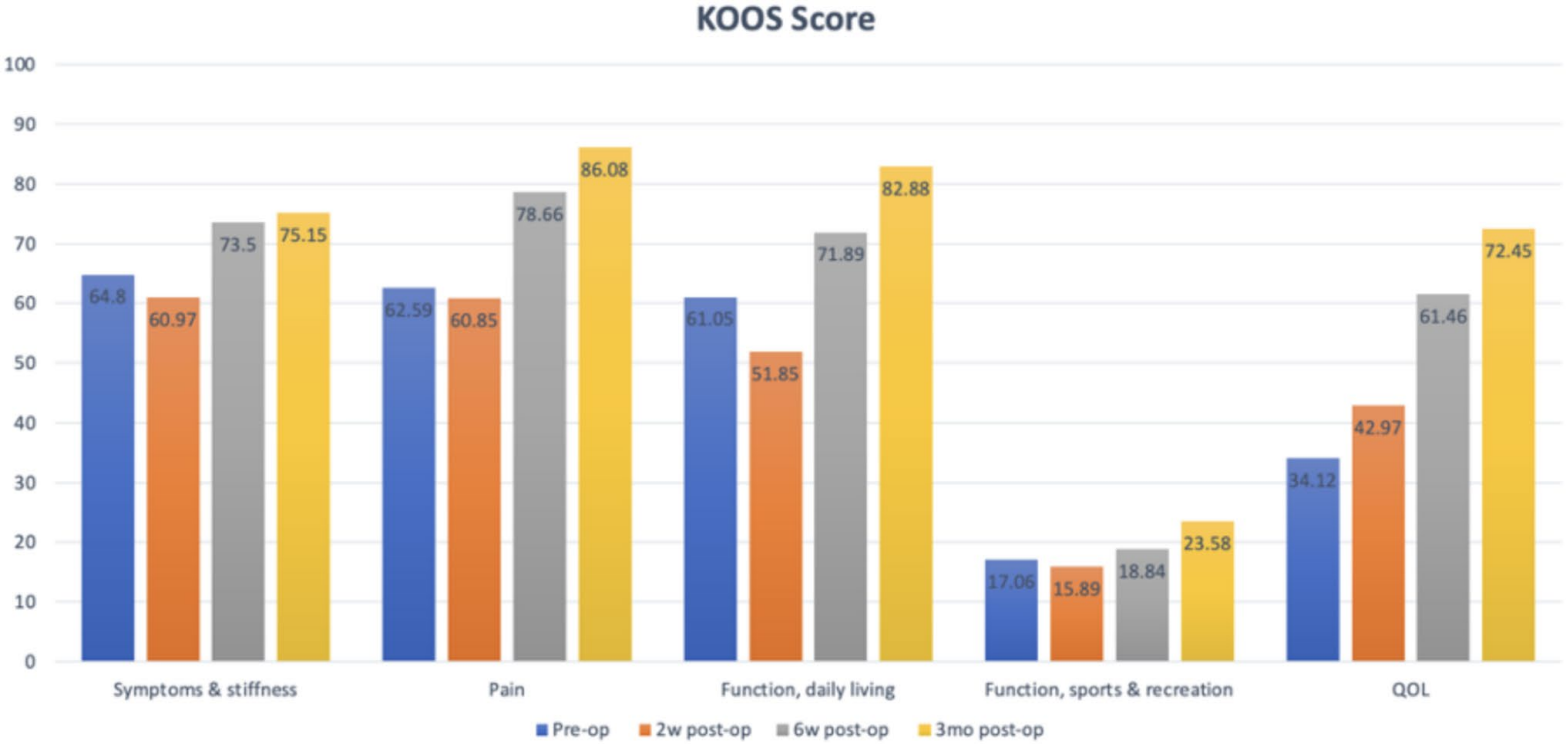
KOOS Score distribution according to subsegment and timeline

KOOS scores showed the initial reductions in Symptoms & stiffness, Pain, and Function (ADL) scores at 2 weeks post-surgery, followed by substantial improvements surpassing pre-operative levels (*p* < 0.0005) by 6 weeks, with continued improvements up to the 3-month assessment. Quality-of-life scores demonstrated improvement as early as 2 weeks post-operatively, and doubling pre-operative scores by 3 months, as illustrated in Fig. 4.

Subgroup analysis based on post-operative HKA axis deviations categorized patients into three groups: neutral axis ± 3 degrees, 3–5 degrees deviation, and greater than 5 degrees deviation. Testing using Kruskal–Wallis to compare the PROM scores against these three groups showed statistically significant difference for the KSS-Functional subsegment, as well as the KOOS subsegment of Function (ADL). Other PROM measures were not statistically significant between the groups. This is captured in Table 2.

**Table 2:** Comparison of PROM measures against HKA axis

PROM category	HKA axis, degrees (°)			Significance, *p* value
	0 ± 3, *N* = *115*	± 3–5, *N* = *36*	> ± 5, *N* = *4*	
KSS				
Objective Knee Indicator	71.65 ± 6.99	70.39 ± 9.45	55.23 ± 20.58	0.307
Symptoms	18.13 ± 4.90	16.61 ± 4.13	14.85 ± 5.90	0.130
Satisfaction	30.64 ± 7.21	29.59 ± 7.67	29.18 ± 1.36	0.652
Expectations	13.31 ± 2.56	12.91 ± 2.69	13.61 ± 0.93	0.543
Functional	45.39 ± 14.96^a^	37.91 ± 15.08^b^	41.02 ± 9.05^ab^	0.042
KOOS				
Symptoms & stiffness	74.00 ± 12.96	71.82 ± 11.62	74.25 ± 5.78	0.611
Pain	80.00 ± 11.32	74.75 ± 11.41	75.33 ± 12.06	0.087
Function, daily living	73.69 ± 12.93	65.94 ± 13.64	73.70 ± 15.25	0.016
Function, sports & recreational	18.54 ± 12.31^a^	19.90 ± 17.41^b^	18.17 ± 2.18^ab^	0.820
Quality of Life	62.33 ± 16.71	58.76 ± 15.92	60.48 ± 12.71	0.697

Different superscript letters (a, b) in a row indicate that there is a significant difference at *p* < 0.05 between the groups using Kruskal–Wallis and Mann–Whitney *U* as post hoc

### Complications

Complications were limited, with four cases of stiffness successfully managed through manipulation under anesthesia and intensified physiotherapy. No cases of superficial skin infection, wound breakdown, prosthetic joint infection, deep vein thrombosis, pulmonary embolism, foot drop, neurovascular injury, or persistent knee swelling requiring drainage were reported.

## Discussion

The MAKO robotic system enables surgeons to adjust bone cuts and optimize the final implant placement with real-time feedback on the resultant hip–knee–ankle (HKA) axis and gap balance throughout the knee's full range of motion. Numerous studies have showed that robotic assistance is able to consistently and accurately achieve mechanical axis restoration within 3 degrees of neutral [9, 12, 14, 24]. However, we believe that correcting the mechanical axis to neutral disregards the significant variability in coronal plane alignment in the population and this overarching goal may in fact result in some dissatisfaction. Hence, the senior author (CHS) now practices a Functional Alignment technique, which utilizes precise bone cuts and limited soft-tissue releases to attain an alignment correction which respects the patient’s inherent anatomy, with equal attention being accorded to joint balance [22, 23]. This technique would not be possible with a conventional TKA technique as it requires real-time feedback with the minute millimeter and 1-degree changes of rotation, which the MAKO system is able to accord the surgeon. The femoral and tibial coronal angles are independently allowed a range of neutral ± 3 degrees, but when taken together, the permissible HKA axis is capped at neutral ± 5 degrees.

With such improvements in the accuracy of implant positioning as well as control over the rotational alignment of the femoral component, patients benefit from better knee kinematics including patella tracking [7, 10, 19]. Enhanced component positioning theoretically leads to less wear, ergo resulting in increased survivorship, which is one aspect that should command significant attention considering that an increasing number of patients undergoing TKA are younger than 65 years old [2, 9].

In this study, the restoration of the hip–knee–ankle (HKA) axis deviated by 0.76 ± 1.9 degrees from the intra-operative plan, which compares favorably to Rossi’s findings using an imageless robotic system, where they reported a differential of 1.2 ± 1.1 degrees [29]. Similarly, Mancino, utilizing a similar imageless robotic system, reported a differential of 1.3 ± 1.0 degrees [30].

The coronal alignment deviations for the femoral and tibial components were 0.08 ± 1.36 and 0.71 ± 1.3 degrees, respectively, from their planned positions intraoperatively. Rossi reported a femoral component deviation of 0.6 ± 0.5 degrees and a tibial component deviation of 0.3 ± 1.8 degrees when assessing for coronal alignment [29]. Hasegawa reported a retrospective study on two cohort of patients who underwent robotic-assisted TKA using two systems—an image-free handheld robotic system (NAVIO) and a radiography-based system (ROSA). In their study, the coronal alignment of the femoral component was 0.36 ± 0.27 degrees for the NAVIO group and 0.29 ± 0.22 degrees for the ROSA group. Tibial component deviations were 0.39 ± 0.32 and 0.55 ± 0.37 degrees for NAVIO and ROSA, respectively [31].

Regarding rotational alignment, deviations averaged 0.39 ± 1.49 degrees in this study, which shows improvement compared to the 1.3-degree average reported by Mahoney et al. who also utilized the Mako robotic system and a post-operative CT scan [32]. Rotational alignment accuracy is challenging to assess in clinical settings without post-operative CT scans, contributing to limited data on this parameter.

Traditionally, gap balancing has relied on surgeon intuition and experience, which can be subjective and variable [7, 8, 25]. Imbalanced gaps lead to suboptimal soft-tissue balance, with a resultant detrimental effect on knee kinematics and an acceleration of polyethylene (PE) wear [25]. Objective assessment and control of gap balance with robotic assistance could potentially lead to a knee that feels more natural, thereby improving patient satisfaction. Studies have shown that patient dissatisfaction often stems from knees that do not feel normal post-surgery. Noble et al. reported that 46% of dissatisfied patients from their cohort revealed that their knees did not feel normal [26], highlighting the importance of achieving a more physiological knee state.

The flexion–extension as well as medial–lateral gaps were well balanced in our cohort of patients. This objective control of gap balance is not possible with conventional jig-based TKA. Our results echo that of Held who demonstrated in his study that the utilization of robotic assistance improved balancing throughout ROM from extension to full flexion as compared to the conventional TKA [33]. However, they also reported that usage of robotic assistance prolonged their average operative time to 127 ± 20 min, which was not the case in our study as average surgical duration was 66.3 ± 12.3 min. Our operative time is aligned to the average time after proficiency (66.8 ± 3.5 min), where Kayani reported a sharp inflexion point and hence a learning curve of seven cases to attain proficiency [34]. 

Improvements in the KSS scores for our patients exceeded the minimum clinically important difference (MCID) of 1.9 points for KSS-Symptoms, 2.2 points for KSS-Satisfaction, and 4.1 points for KSS-Functional by 6 weeks post-op [27]. Monticone et al. reported the MCID of KOOS scores for patients who underwent a TKA being 10.7 points for Symptoms, 16.7 for Pain, 18.4 for ADL, 12.5 for Sports, and 15.6 for Quality of Life [28]. Our cohort of patients exceeded the MCID for KOOS-Pain and KOOS-ADL by 3 months post-op and KOOS-QOL as soon as 6 weeks post-TKA. Achieving and exceeding the MCID so early in their rehabilitation reaffirm that robotic-TKA can confer an expedited recovery ergo potentially translating into improved satisfaction.

Robotic assistance also promises cost savings through reduced instrument trays, shorter operating times, and fewer intra-operative complications such as inadvertent soft-tissue injury [9]. Cumulative robotic experience has shown no negative impact on accuracy of implant positioning, limb alignment, and joint line restoration [13]. As such, increased robotic-assisted TKA would hopefully result in better outcomes and implant survivorship, indirectly circumventing the potential costs incurred for revision surgery.

This study is not without limitations. This is a short-term report after 3 months of reviews. While we acknowledge that the short follow-up duration might not be able to fully capture the full extent of patient recovery and complications, we are writing this report to highlight the degree of accuracy and balance control that robotic assistance allows us to achieve and how that translates to good early outcomes thus far. As this is a preliminary report, we are extremely encouraged by the results attained and we will be publishing further studies reporting their long-term outcomes in the future. Next, we did not subject our patients to post-operative long-limb lateral view radiographs. As such, we were unable to ascertain the accuracy of implant positioning in the sagittal plane. We understand that some studies utilize navigation systems to determine accuracy of implant placement; however, their use may incur additional costs and prolong operating room time, factors we sought to minimize for our patients.

## Conclusion

Robotic-assisted TKA offers precise control in the accuracy of implant positioning, gap balancing, and attainment of the desired alignment, with significant reduction in the risk of peri-articular soft-tissue trauma, and is resulting in excellent early post-operative outcomes. Future studies with longer follow-up periods are warranted to further elucidate the full extent of benefits of this technology.

## Data Availability

The datasets used and/or analyzed during the current study are available from the corresponding author on reasonable request.

## References

[1] Gunaratne R, Pratt DN, Banda J, Fick DP, Khan RJK, Robertson BW (2017) Patient dissatisfaction following total knee arthroplasty: a systematic review of the literature. J Arthroplasty 32(12):3854–3860. 10.1016/j.arth.2017.07.021 28844632 10.1016/j.arth.2017.07.021

[2] Smith AF, Eccles CJ, Bhimani SJ et al (2021) Improved patient satisfaction following robotic-assisted total knee arthroplasty. J Knee Surg 34(7):730–738. 10.1055/s-0039-1700837 31731324 10.1055/s-0039-1700837

[3] Kayani B, Konan S, Tahmassebi J, Pietrzak JRT, Haddad FS (2018) Robotic-arm assisted total knee arthroplasty is associated with improved early functional recovery and reduced time to hospital discharge compared with conventional jig-based total knee arthroplasty: a prospective cohort study. Bone Joint J. 100-B(7):930–937. 10.1302/0301-620X.100B7.BJJ-2017-1449.R1 29954217 10.1302/0301-620X.100B7.BJJ-2017-1449.R1 PMC6413767

[4] Decking J, Theis C, Achenbach T, Roth E, Nafe B, Eckardt A (2004) Robotic total knee arthroplasty: the accuracy of CT-based component placement. Acta Orthop Scand 75(5):573–579. 10.1080/00016470410001448 15513489 10.1080/00016470410001448

[5] Kayani B, Konan S, Pietrzak JRT, Haddad FS (2018) Iatrogenic bone and soft tissue trauma in robotic-arm assisted total knee arthroplasty compared with conventional jig-based total knee arthroplasty: a prospective cohort study and validation of a new classification system. J Arthroplasty 33(8):2496–2501. 10.1016/j.arth.2018.03.042 29699827 10.1016/j.arth.2018.03.042

[6] King CA, Jordan M, Bradley AT, Wlodarski C, Tauchen A, Puri L (2022) Transitioning a practice to robotic total knee arthroplasty is correlated with favorable short-term clinical outcomes-a single surgeon experience. J Knee Surg 35(1):78–82. 10.1055/s-0040-1712984 32544972 10.1055/s-0040-1712984

[7] Mason JB, Fehring TK, Estok R, Banel D, Fahrbach K (2007) Meta-analysis of alignment outcomes in computer-assisted total knee arthroplasty surgery. J Arthroplasty 22(8):1097–1106. 10.1016/j.arth.2007.08.001 18078876 10.1016/j.arth.2007.08.001

[8] Gustke KA, Golladay GJ, Roche MW, Elson LC, Anderson CR (2014) A new method for defining balance: promising short-term clinical outcomes of sensor-guided TKA. J Arthroplasty 29(5):955–960. 10.1016/j.arth.2013.10.020 24269069 10.1016/j.arth.2013.10.020

[9] Khlopas A, Sodhi N, Sultan AA, Chughtai M, Molloy RM, Mont MA (2018) Robotic arm-assisted total knee arthroplasty. J Arthroplasty 33(7):2002–2006. 10.1016/j.arth.2018.01.060 29506926 10.1016/j.arth.2018.01.060

[10] Liow MHL, Goh GS, Wong MK, Chin PL, Tay DK, Yeo SJ (2017) Robotic-assisted total knee arthroplasty may lead to improvement in quality-of-life measures: a 2-year follow-up of a prospective randomized trial. Knee Surg Sports Traumatol Arthrosc 25(9):2942–2951. 10.1007/s00167-016-4076-3 27017214 10.1007/s00167-016-4076-3

[11] Mannan A, Vun J, Lodge C, Eyre-Brook A, Jones S (2018) Increased precision of coronal plane outcomes in robotic-assisted total knee arthroplasty: a systematic review and meta-analysis. Surgeon 16(4):237–244. 10.1016/j.surge.2017.12.003 29439922 10.1016/j.surge.2017.12.003

[12] Marchand RC, Sodhi N, Khlopas A et al (2017) Patient satisfaction outcomes after robotic arm-assisted total knee arthroplasty: a short-term evaluation. J Knee Surg 30(9):849–853. 10.1055/s-0037-1607450 29029352 10.1055/s-0037-1607450

[13] Zhang J, Ndou WS, Ng N et al (2022) Robotic-arm assisted total knee arthroplasty is associated with improved accuracy and patient reported outcomes: a systematic review and meta-analysis. Knee Surg Sports Traumatol Arthrosc 30(8):2677–2695. 10.1007/s00167-021-06464-4 33547914 10.1007/s00167-021-06464-4 PMC9309123

[14] Song EK, Seon JK, Yim JH, Netravali NA, Bargar WL (2013) Robotic-assisted TKA reduces postoperative alignment outliers and improves gap balance compared to conventional TKA. Clin Orthop Relat Res 471(1):118–126. 10.1007/s11999-012-2407-3 22669549 10.1007/s11999-012-2407-3 PMC3528918

[15] Choong PF, Dowsey MM, Stoney JD (2009) Does accurate anatomical alignment result in better function and quality of life? Comparing conventional and computer-assisted total knee arthroplasty. J Arthroplasty 24(4):560–569. 10.1016/j.arth.2008.02.018 18534397 10.1016/j.arth.2008.02.018

[16] Kurtz SM, Lau E, Ong K, Zhao K, Kelly M, Bozic KJ (2009) Future young patient demand for primary and revision joint replacement: national projections from 2010 to 2030. Clin Orthop Relat Res 467(10):2606–2612. 10.1007/s11999-009-0834-6 19360453 10.1007/s11999-009-0834-6 PMC2745453

[17] Khlopas A, Sodhi N, Hozack WJ et al (2020) Patient-reported functional and satisfaction outcomes after robotic-arm-assisted total knee arthroplasty: early results of a prospective multicenter investigation. J Knee Surg 33(7):685–690. 10.1055/s-0039-1684014 30959541 10.1055/s-0039-1684014

[18] Johnson DS, Ryan WG, Smith RB (2004) Does the Lachman testing method affect the reliability of the international knee documentation committee (IKDC) form? Knee Surg Sports Traumatol Arthrosc 12(3):225–228. 10.1007/s00167-003-0475-3 14691622 10.1007/s00167-003-0475-3

[19] Miller MC, Berger RA, Petrella AJ, Karmas A, Rubash HE (2001) Optimizing femoral component rotation in total knee arthroplasty. Clin Orthop Relat Res 392:38–45. 10.1097/00003086-200111000-00005 10.1097/00003086-200111000-00005 11716411

[20] Pallant J (2007) SPSS survival manual, a step by step guide to data analysis using SPSS for windows, 3rd edn. McGraw Hill, Sydney, pp 179–200

[21] Field A (2009) Discovering statistics using SPSS, 3rd edn. SAGE publications Ltd, London, p 822

[22] Shatrov J, Battelier C, Sappey-Marinier E, Gunst S, Servien E, Lustig S (2022) Functional alignment philosophy in total knee arthroplasty - rationale and technique for the varus morphotype using a CT based robotic platform and individualized planning. SICOT J. 8:11. 10.1051/sicotj/2022010 35363136 10.1051/sicotj/2022010 PMC8973302

[23] Clark G, Steer R, Wood D (2023) Functional alignment achieves a more balanced total knee arthroplasty than either mechanical alignment or kinematic alignment prior to soft tissue releases. Knee Surg Sports Traumatol Arthrosc 31(4):1420–1426. 10.1007/s00167-022-07156-3 36116071 10.1007/s00167-022-07156-3 PMC10050049

[24] Bellemans J, Vandenneucker H, Vanlauwe J (2007) Robot-assisted total knee arthroplasty. Clin Orthop Relat Res 464:111–116. 10.1097/BLO.0b013e318126c0c0 17563698 10.1097/BLO.0b013e318126c0c0

[25] Shatrov J, Parker D (2020) Computer and robotic - assisted total knee arthroplasty: a review of outcomes. J Exp Orthop 7(1):70. 10.1186/s40634-020-00278-y 32974864 10.1186/s40634-020-00278-y PMC7516005

[26] Noble PC, Conditt MA, Cook KF, Mathis KB (2006) The John insall award: patient expectations affect satisfaction with total knee arthroplasty. Clin Orthop Relat Res 452:35–43. 10.1097/01.blo.0000238825.63648.1e 16967035 10.1097/01.blo.0000238825.63648.1e

[27] Nishitani K, Yamamoto Y, Furu M et al (2019) The minimum clinically important difference for the Japanese version of the new knee society score (2011KSS) after total knee arthroplasty. J Orthop Sci 24(6):1053–1057. 10.1016/j.jos.2019.09.001 31543424 10.1016/j.jos.2019.09.001

[28] Monticone M, Ferrante S, Salvaderi S, Motta L, Cerri C (2013) Responsiveness and minimal important changes for the knee injury and osteoarthritis outcome score in subjects undergoing rehabilitation after total knee arthroplasty. Am J Phys Med Rehabil 92(10):864–870. 10.1097/PHM.0b013e31829f19d8 23900017 10.1097/PHM.0b013e31829f19d8

[29] Rossi SMP, Sangaletti R, Perticarini L, Terragnoli F, Benazzo F (2023) High accuracy of a new robotically assisted technique for total knee arthroplasty: an in vivo study. Knee Surg Sports Traumatol Arthrosc 31(3):1153–1161. 10.1007/s00167-021-06800-8 34981162 10.1007/s00167-021-06800-8 PMC8723813

[30] Mancino F, Rossi SMP, Sangaletti R, Caredda M, Terragnoli F, Benazzo F (2024) Increased accuracy in component positioning using an image-less robotic arm system in primary total knee arthroplasty: a retrospective study. Arch Orthop Trauma Surg 144(1):393–404. 10.1007/s00402-023-05062-y 37755480 10.1007/s00402-023-05062-y

[31] Hasegawa M, Tone S, Naito Y, Sudo A (2024) Comparison of accuracy and early outcomes in robotic total knee arthroplasty using NAVIO and ROSA. Sci Rep 14(1):3192. 10.1038/s41598-024-53789-4 38326363 10.1038/s41598-024-53789-4 PMC10850152

[32] Mahoney O, Kinsey T, Sodhi N et al (2022) Improved component placement accuracy with robotic-arm assisted total knee arthroplasty. J Knee Surg 35(3):337–344. 10.1055/s-0040-1715571 32869232 10.1055/s-0040-1715571

[33] Held MB, Grosso MJ, Gazgalis A et al (2021) Improved compartment balancing using a robot-assisted total knee arthroplasty. Arthroplast Today. 7:130–134. 10.1016/j.artd.2020.12.022 33553538 10.1016/j.artd.2020.12.022 PMC7850935

[34] Kayani B, Konan S, Huq SS, Tahmassebi J, Haddad FS (2019) Robotic-arm assisted total knee arthroplasty has a learning curve of seven cases for integration into the surgical workflow but no learning curve effect for accuracy of implant positioning. Knee Surg Sports Traumatol Arthrosc 27(4):1132–1141. 10.1007/s00167-018-5138-5 30225554 10.1007/s00167-018-5138-5 PMC6435632

